# Bovine Serum Albumin-Functionalized Hyperbranched Polyamidoamine Dendrimers (BSA@PAMAM) for GSH-Responsive Bacteriostasis

**DOI:** 10.1021/acsomega.5c09261

**Published:** 2026-01-16

**Authors:** Yu Fu, Yixuan Ren, Shufen Xiao, Jian Chen, Xingling Liu

**Affiliations:** † Department of Pharmacy, Third Affiliated Hospital of Sun Yat-sen University, Guangzhou 510630, China; ‡ School of Chemistry and Chemical Engineering, 12518 Hunan University of Science and Technology, Xiangtan, Hunan 411201, China

## Abstract

Traditional antibiotics (such as levofloxacin) are mainly administered orally or via injection. However, these delivery methods struggle to maintain an optimal drug concentration, leading to low bioavailability and potential toxic side effects. Therefore, this study proposes a controlled-release antibiotic delivery system to achieve on-demand drug administration. Specifically, the system utilizes the hydrophobic cavities of PAMAM to efficiently load antibiotics and modifies its surface with hydrophilic BSA via coupling to enhance nanodrug stability during systemic circulation. BSA is conjugated to PAMAM using an *N*-hydroxysuccinimide active ester containing disulfide bonds, enabling drug release through disulfide bond cleavage in response to glutathione (GSH). Characterization techniques, including nuclear magnetic resonance, Fourier transform infrared spectroscopy, dynamic light scattering, contact angle measurement, and thermogravimetric analysis, confirmed the successful construction of the nanodrug. In vitro drug release experiments demonstrated the nanodrug’s responsiveness to GSH. Additionally, antibacterial assays showed that the BSA@PAMAM nanodrug loaded with levofloxacin exhibited enhanced antibacterial effects in a GSH environment, indicating that this system can effectively regulate antibiotic release, optimize drug administration, improve therapeutic efficacy, and reduce adverse effects.

## Graphical abstract

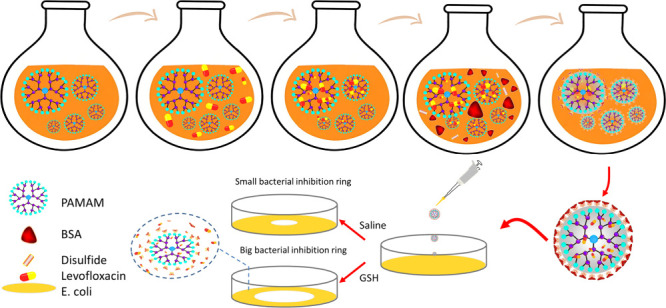

## 1 Introduction

In clinical applications, the selection of antibiotics must be tailored to specific pathogens to ensure efficacy and specificity.^1^ Antimicrobial agents primarily inhibit or eliminate pathogens by interfering with bacterial metabolism, growth, and reproduction. To achieve optimal therapeutic effects, it is necessary to maintain an adequate drug concentration in the bloodstream, making appropriate and sufficient dosing essential.^2^ Additionally, antibiotic treatment must be sustained for a certain period to ensure complete eradication of pathogens and reduce the risk of resistance.^3^ However, prolonged use may lead to adverse reactions, potentially harming overall health.^4^ Therefore, antibiotic therapy should be discontinued as soon as the infection is controlled to minimize drug-related side effects. Given these challenges associated with antibiotic use, the development of controlled-release nanodrug is of great significance.^5^ By regulating antibiotic release through nanodrug systems, it is possible to achieve on-demand drug delivery, maintain effective concentrations, and optimize dosing regimens.^6^ This approach ensures therapeutic efficacy while reducing antibiotic overuse and associated adverse effects.^7^ A well-designed drug release strategy can help prevent disruptions to the gut microbiota, lower the risk of resistance, and minimize damage to liver and kidney function, the hematopoietic system, and the nervous system.^8^ Bacterial infections can induce inflammation, during which cells may upregulate the expression of GSH to counteract oxidative stress caused by the inflammation. This helps detoxify and eliminate inflammatory mediators, or directly neutralizes ROS (reactive oxygen species) by activating antioxidant responses through pathways like Nrf2, thereby upregulating the synthesis of GSH and increasing its intracellular levels.^9^ At the same time, the concentration of GSH within cells is much higher than that in the blood. Therefore, GSH-responsive drug release can be used to regulate the release of therapeutic agents. By utilizing the high concentration of GSH in infected cells, drugs can be intelligently released in response to the intracellular environment, allowing for precise targeted therapy of intracellular infections.^10,11^ Thus, the construction of intelligent antibiotic delivery systems presents a viable strategy for enhancing the safety and efficacy of antimicrobial therapy.

In 1978, Vögtle et al. first reported dendrimers (also known as “cascade molecules”), whose unique molecular architecture distinguishes them from traditional linear, cross-linked, or simple branched polymers.^12^ Dendrimers extend outward from a central core, forming a well-defined cascade of branches and possessing a large number of terminal functional groups on their surface. This structural characteristic endows them with unique physicochemical properties and multivalent cooperativity, enabling outstanding performance in various applications. Due to their amphiphilic nature, small amphiphilic dendrimers can self-assemble into larger supramolecular structures through noncovalent weak interactions such as van der Waals forces, hydrogen bonding, and electrostatic interactions.^13^ This self-assembly capability further expands their potential applications in the biomedical field, particularly in drug delivery, diagnostics, and therapeutics.^14^ Amphiphilic dendritic structures can form internal cavities within supramolecular systems for physical drug encapsulation, while their abundant surface functional groups allow for drug adsorption or chemical conjugation, enhancing drug delivery stability and targeting efficiency. Among them, PAMAM dendrimers, characterized by their amide backbone and rich internal and terminal amine functional groups, exhibit excellent biocompatibility and have become one of the most extensively studied dendrimers. The PAMAM structure can be tailored in terms of size, shape, and surface modifications to meet the requirements of specific biomedical applications, offering great potential for precision drug delivery and targeted therapy.

In addition, the interactions between biomedical nanomaterials and biomacromolecules, cells, and biological systems are crucial for the development of sustainable nanotechnology.^15^ Studies have shown that exogenous compounds often bind to proteins upon entering biological fluids (such as plasma), and the interactions between nanoparticles (NPs) and proteins can significantly influence their in vivo transport and biological fate. Nanoparticles may be phagocytosed by the reticuloendothelial system (RES) or adsorb proteins in the bloodstream, thereby affecting their stability and targeting ability. A major challenge faced by nanoantibiotic drugs is their stability in circulation and limited penetration into infected tissues. To enhance therapeutic efficacy, drugs must remain stable in the bloodstream while efficiently penetrating infected tissues to achieve precise targeted therapy. Compared with traditional small-molecule drugs, nanoparticles, due to their larger size, can accumulate at infection sites through the enhanced permeability and retention (EPR) effect, thereby increasing drug concentration and prolonging therapeutic action.^16^ Optimizing the physical and chemical properties of nanoparticles, such as particle size, shape, and surface modifications, is a key strategy to improve their biocompatibility and therapeutic effects. By tuning the hydrophobicity, hydrophilicity, and surface functionalization of nanomaterials, their in vivo behavior can be effectively improved. Additionally, leveraging the controlled synthesis characteristics of dendritic polymers, chemical modifications of terminal groups or the construction of covalent supramolecular dendritic polymers via self-assembly provide feasible solutions for enhancing the stability and targeting efficiency of nanoantibiotic drugs.^17^

Albumin is widely distributed in the human body and has been extensively used in nanoparticle surface modification research in recent years due to its excellent biodegradability, nonimmunogenicity, and outstanding water solubility. Rich in hydrophilic residues, albumin exhibits good water solubility and strong affinity for various proteins in the bloodstream. Compared with inorganic materials or synthetic polymers, albumin-based nanoparticles demonstrate superior biodegradability and physiological stability, which not only prolongs the circulation time of nanodrugs in vivo but also enhances their accumulation at infection sites.^18^ In the strategy of PAMAM and BSA complexation, existing literature primarily reports two main approaches: physical adsorption and covalent coupling. In the case of physical adsorption, Pathak et al. covalently modified mannose onto the surface of PAMAM and utilized electrostatic interactions, hydrogen bonding, and hydrophobic interactions to achieve physical binding with BSA.^19^ Chanphai et al. discovered that PAMAM-G4 could adsorb onto HSA/BSA via hydrophobic interactions and hydrogen bonds, demonstrating its potential as a drug carrier.^20^ Mandeville and Zhang’s research teams systematically investigated the physical adsorption mechanism between PAMAM and BSA, analyzing the structural changes of BSA during the complexation process.^21,22^ In terms of covalent coupling, Kuan et al. employed click chemistry by introducing an azide group on HSA and an alkyne group on PAMAM, utilizing the azide–alkyne cycloaddition reaction to achieve efficient covalent linkage between the two.^23^ Their study also highlighted that noncovalent interactions such as hydrophobic, electrostatic, and hydrogen bonding played an auxiliary role in the system, and that BSA coating effectively reduced the cytotoxicity of PAMAM.^24^ In conclusion, the complexation of PAMAM with BSA can be achieved either through intermolecular interactions for physical adsorption or through covalent bonds (especially via click chemistry) to form a more stable complex structure. The latter approach offers advantages in terms of coupling efficiency and structural control. During the conjugation of albumin with PAMAM dendrimers, the abundant amine functional groups of PAMAM provide effective pathways for nanoparticle conjugation. The coupling between albumin and PAMAM enhances the structural stability of nanoparticles in blood circulation while improving their biocompatibility and permeability. Compared to traditional covalent modification strategies, this method is simple to operate, avoids complex dendrimer synthesis processes, and imparts excellent colloidal stability to nanoparticles.^25,26^ Thus, albumin-modified PAMAM not only improves the stability of nanodrugs in circulation but also enhances their tissue penetration, making it an ideal strategy for optimizing nanodrug delivery systems.

This study leverages the unique radial branching structure of dendrimers to provide abundant drug-loading sites for encapsulating levofloxacin, while utilizing albumin’s stability in blood circulation to enhance biocompatibility and delivery performance through surface modification. Specifically, an *N*-hydroxysuccinimide (NHS)-activated disulfide compound was used as a linker between albumin and the dendrimer, enabling rapid and efficient albumin modification via the high-efficiency coupling reaction between NHS esters and amine groups, while encapsulating levofloxacin within the internal cavities of the dendrimer (Scheme 1). Furthermore, since disulfide bonds can undergo reductive cleavage in the presence of GSH in vivo, albumin detaches from the dendrimer surface, exposing the dendrimer core and facilitating levofloxacin release, thereby achieving GSH-responsive controlled drug release.^27^ Drug release experiments in a GSH environment were conducted to verify the controlled release capability of the levofloxacin-loaded BSA@PAMAM nanodrug, and antibacterial zone of inhibition assays were performed to evaluate its antibacterial efficacy against *Escherichia coli* in the presence of GSH. This strategy holds promise for improving the precision and therapeutic efficacy of antibiotic delivery while reducing the risk of resistance, offering new insights for the development of intelligent antibiotic delivery systems.

**Scheme 1:**
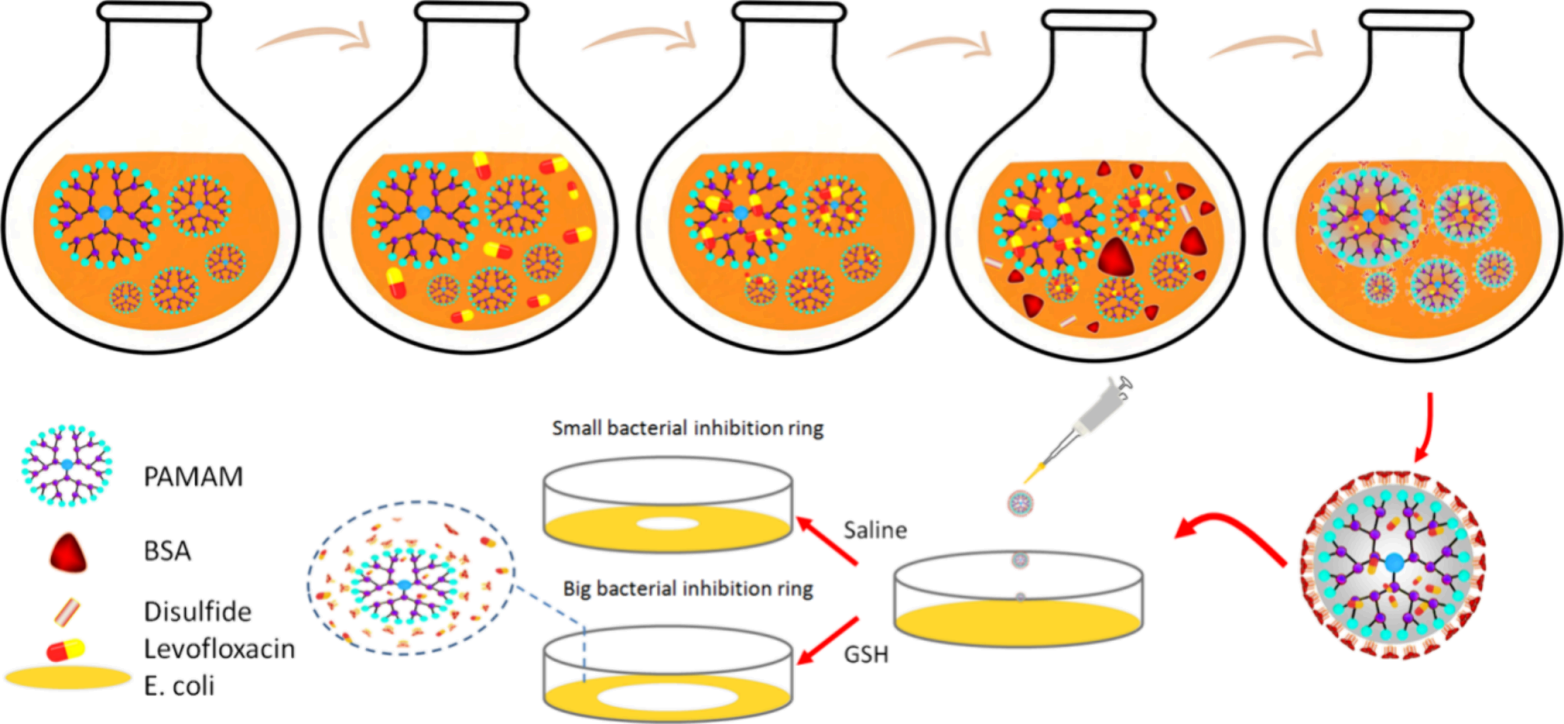
Schematic Illustration of the Assembly of Levofloxacin-Loaded BSA@PAMAM and the Mechanism of GSH-Responsive Drug Release for Antibacterial Action

## 2 Experimental Section

### 2.1 Material Preparation and Characterization

3,3′-Disulfanediyldipropionic acid, NHS, and 1-ethyl-3-(3-dimethylaminopropyl)­carbodiimide hydrochloride (EDCI) were purchased from tansoole (Shanghai, China), while BSA, GSH, and PAMAM-G4 were obtained from Shanghai Yuanye Bio-Technology Co., Ltd. (China). ^1^H NMR and ^13^C NMR spectra were analyzed using a Bruker 400 MHz NMR spectrometer (AVANCE NEO 400 M). FTIR spectra were recorded using a Thermo Nicolet iS50 FTIR spectrometer. Particle size distribution was measured using a dynamic light scattering (DLS) instrument (Zetasizer Pro, Malvern Panalytical Ltd.). The hydrophilicity and hydrophobicity of the materials were characterized using a contact angle goniometer (Tianyan TY-SDJ03, China).

### 2.2 Preparation of NHS-Activated Disulfide Compound

A mixture of 3,3′-disulfanediyldipropionic acid (0.5 g, 2.37 mmol), NHS (0.6 g, 5.23 mmol), and EDCI (1.0 g, 5.23 mmol) was dissolved in 20 mL of dichloromethane. The solution was stirred at room temperature overnight. After the reaction was complete, the solvent was removed by rotary evaporation, yielding a solid residue. The crude product was purified by silica gel column chromatography using ethyl acetate:methanol (50:1) as the mobile phase. The target fractions were collected, and the solvent was removed by rotary evaporation, yielding a white solid (0.68 g) with a yield of 71%.

### 2.3 Preparation of Levofloxacin-Loaded BSA@PAMAM

A total of 0.1 g of PAMAM powder was dissolved in 20 mL of deionized water and stirred until fully dissolved. Then, 0.1 g of levofloxacin was added to the solution and stirred for 24 h, allowing levofloxacin to be loaded into the hydrophobic cavities of PAMAM via hydrophobic interactions. Subsequently, 0.2 g of BSA and 0.05 g of NHS-activated disulfide compound were added sequentially to the solution. The NHS ester-mediated coupling reaction facilitated the binding of BSA to PAMAM, forming a hydrophilic layer on the PAMAM surface while encapsulating the loaded levofloxacin. The solution was further stirred for 12 h to ensure complete reaction. The resulting reaction mixture was transferred into a dialysis bag (MWCO: 100 kDa) and dialyzed against deionized water for 24 h, with the dialysis medium replaced every 6 h to remove unbound BSA and unencapsulated levofloxacin. After dialysis, the sample was lyophilized to obtain 0.29 g of white powder, representing the levofloxacin-loaded BSA@PAMAM complex, which was used for subsequent characterization and performance evaluation.

### 2.4 Determination of Drug Loading and Encapsulation Efficiency

Weigh 0.1 g of freeze-dried levofloxacin-loaded BSA@PAMAM powder and dissolve it in 100 mL of 10 mM GSH solution. Perform ultrasonication for 30 min, then let the solution stand for another 30 min while stirring continuously overnight to ensure complete drug release. Take a portion of the solution and centrifuge it using an Amicon centrifugal filter tube (MWCO: 3 kDa) to collect the filtrate. The absorbance of levofloxacin in the filtrate is measured using UV–vis spectroscopy with a maximum detection wavelength of 290 nm, and the drug loading is calculated by comparing it with the standard calibration curve (Figure S1, Supporting Information), resulting in a drug loading of 15.2% and a encapsulation efficiency of 44%. Similarly, we used the same method to test the drug loading and encapsulation efficiency of levofloxacin-loaded PAMAM powder. The measured results were 41% of drug loading content and 49% of encapsulation efficiency. The calculation formulas used were as follows: Drug Loading Content = (Weight of the loaded drug in nanocarrier/Total weight of nanocarrier and the loaded drug) × 100%; Encapsulation Efficiency = (Weight of the loaded drug/Total initial drug) × 100%.

### 2.5 In Vitro Drug Release Study

To evaluate the drug release behavior, weigh 0.1 g of freeze-dried levofloxacin-loaded BSA@PAMAM powder and dissolve it separately in 5 mL of physiological saline (pH = 7.4) solution and 5 mL of physiological saline (pH = 7.4) + 5 mM GSH solution. Transfer the prepared solutions into dialysis bags (MWCO: 3 kDa) and immerse the dialysis bags in 95 mL of the corresponding release medium (physiological saline or physiological saline +5 mM GSH). The samples are incubated in a thermostatic shaker with continuous shaking. At predetermined time points, withdraw 1 mL of the dialysate for analysis. The concentration of levofloxacin in the dialysate is determined using UV–vis spectroscopy to assess drug release behavior. The volume of the release medium is maintained constant by replenishing an equal volume of fresh medium after each sampling to ensure stable experimental conditions. This experimental design evaluates the release behavior of BSA@PAMAM-loaded levofloxacin nanoparticles under physiological conditions (physiological saline) and GSH-triggered conditions (physiological saline + GSH), providing reference data for antibacterial applications.

### 2.6 Cytotoxicity Analysis Experiment

Add 100 μL of cell suspension (HEK 293T) to each well of a 96-well plate, with 5000 cells seeded per well. Each experimental group is set up with five replicate wells. Incubate the cell plate in a 37 °C, 5% CO_2_ incubator for 24 h to ensure proper cell adhesion. Remove the original culture medium from each well and add fresh medium containing different concentrations of the blank nanodrug to be tested. An equal volume of nanodrug-free medium is added as a control. The cell culture plate is then placed back into the 37 °C, 5% CO_2_ incubator for another 24-h incubation. Next, add 10 μL of CCK-8 (Beyotime Biotechnology, China) reagent to each well. Gently shake the plate to ensure even distribution of the CCK-8 reagent while avoiding bubble formation, which may affect subsequent optical density (OD) readings. Incubate the plate in the incubator for an additional 2 h. The absorbance (OD value) of each well is measured at 450 nm using a microplate reader (Varioskan LUX, Thermo Scientific). Record the data and calculate the relative cell viability. The cell survival rate is determined using the following formula: Relative cell viability (%) = (OD value of experimental group/OD value of control group) × 100%.

### 2.7 Antibacterial Effect of Inhibition Assay

Weigh the required amounts of tryptone, yeast extract, and NaCl, and place them in a beaker. Add approximately two-thirds of the final volume of distilled water and stir with a glass rod until fully dissolved. Adjust the pH to 7.2 using a 1 mol/L NaOH solution. Transfer the solution to a graduated cylinder and add water to reach the target volume. Add an appropriate amount of agar, heat until completely melted, and compensate for any water loss due to evaporation. Dispense the medium into test tubes or culture bottles, cover, and wrap them properly. Sterilize by autoclaving at 121 °C and 100 kPa for 20 min. Culture *Escherichia coli* (DH5α) to an appropriate concentration. Dilute the bacterial suspension to the required experimental concentration to ensure result comparability. Pour the sterilized culture medium into sterile Petri dishes and allow it to solidify naturally. Evenly spread the diluted bacterial suspension onto the agar plate surface, ensuring uniform distribution. Incubate the plates at an appropriate temperature until the bacteria reach the logarithmic growth phase. Using sterile filter paper discs, apply the test drug solution onto the discs and gently place them at the center of the agar plates. Incubate the plates in a constant-temperature incubator for 24 h. Observe and measure the diameter of the inhibition zones to assess the antibacterial activity of the different nanodrugs. Record the inhibition zone sizes for each experimental group to evaluate the nanodrugs’ antibacterial effectiveness. At the same time, *Escherichia coli* in the logarithmic growth phase was cultured and then separately added to saline and GSH solutions. The test drug solution was added to the corresponding culture dishes. At different time points, bacterial solutions were mixed with a dead bacteria staining reagent (Shanghai Yuanye Bio-Technology Co., Ltd.) and subjected to fluorescence imaging analysis.

## 3 Results and Discussion

### 3.1 Preparation and Characterization of Levofloxacin-Loaded BSA@PAMAM

This study is based on a functionalized nanodrug system utilizing BSA@PAMAM, where 3,3′-dithiodipropionic acid serves as a coupling agent. The controlled drug release is achieved through GSH-induced disulfide bond cleavage. Additionally, the formation of efficient ester bond coupling via EDC/NHS activation ensures stable conjugation between BSA and PAMAM, enhancing its biocompatibility and drug-loading capacity. The chemical structure of the synthesized NHS-activated disulfide compound was characterized by NMR, including ^1^H NMR and ^13^C NMR (Figure 1). The results are as follows: ^1^H NMR (400 MHz, CDCl_3_, δ, ppm): 3.09 (t, 4H, *J* = 6 Hz, −CO–CH_2_–CH_2_–S−), 3.03 (t, 4H, *J* = 6 Hz, −CO–CH_2_–CH_2_–S−), 2.85 (s, 8H, −CO–CH_2_–CH_2_–CO−).^13^C NMR (101 MHz, CDCl_3_, δ, ppm): 168.97 (−CH_2_–CO–N−), 167.06 (−O–CO−), 32.26 (−CO–CH_2_–CH_2_–S−), 31.11 (−CO–CH_2_–CH_2_–S−), 25.6 (−CO–CH_2_–CH_2_–CO−). The NMR results confirm that the synthesized coupling agent has a well-defined structure, with key chemical shift signals matching the expected values, verifying its successful synthesis. Future research could focus on further optimizing the structure of the conjugated compounds to enhance drug release precision and in vivo stability, thereby advancing their applications in drug delivery systems. A disulfide compound with NHS-activated esters at both ends (NHS-activated disulfide compound) can directly utilize the abundant amine groups on the surfaces of PAMAM and BSA for efficient coupling, without the need for prior modification. This method not only avoids complex chemical treatments, purification steps, and the potential structural damage and chemical residues that could arise, but also achieves more stable BSA coating through covalent bonds, which are stronger than noncovalent interactions like hydrophobic forces. Furthermore, the disulfide bonds in this compound serve as GSH-responsive sites, providing a foundation for the controlled release of the drug.^28^

**Figure 1:**
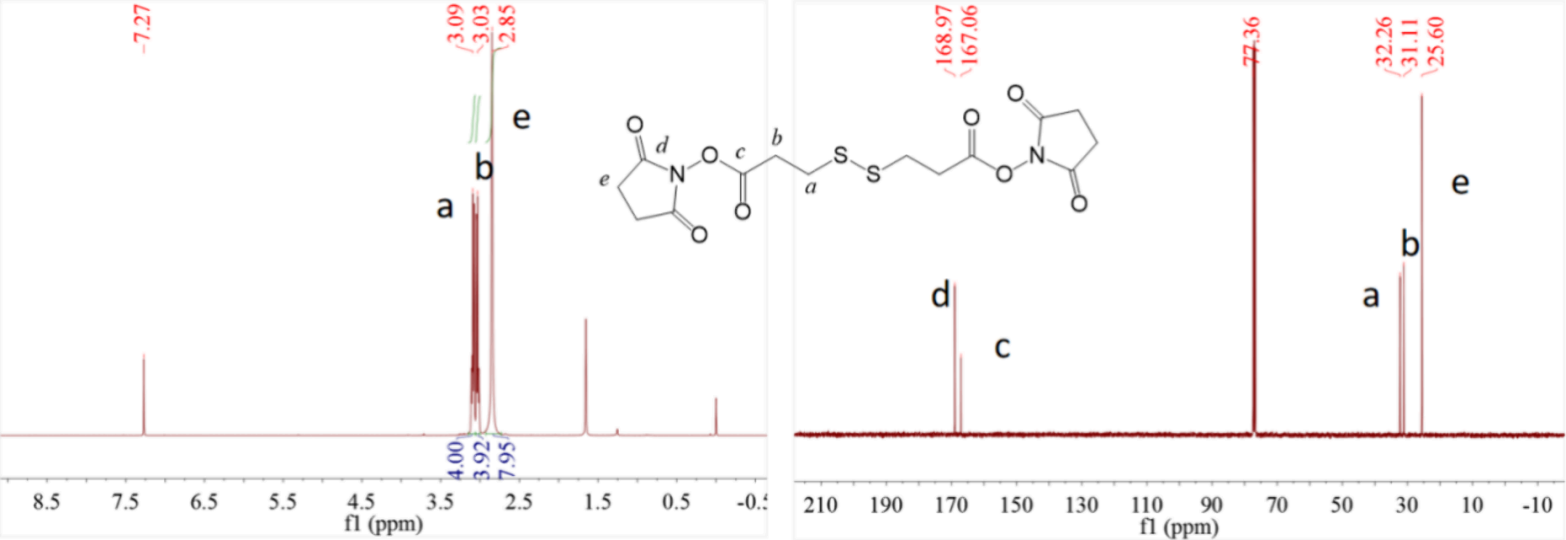
^1^H NMR and ^13^C NMR spectra of NHS-activated disulfide compound.

FTIR was employed to investigate the structural formation process of BSA@PAMAM nanodrug. As shown in Figure 2, PAMAM exhibits a characteristic C–N stretching vibration peak at 1023 cm^–1^. The peaks at 1652 and 1541 cm^–1^ correspond to N–H and C=O bending and stretching vibrations, respectively. The peaks at 2947 and 2843 cm^–1^ correspond to C–H stretching vibrations, while the peaks at 3297 cm^–1^ represents asymmetric and symmetric NH_2_ stretching vibrations. Levofloxacin exhibits benzene ring C–H out-of-plane bending vibrations at 709 and 798 cm^–1^, an asymmetric C–O–C stretching vibration at 978 cm^–1^, C–F stretching vibrations at 1050 and 1292 cm^–1^. For levofloxacin-loaded PAMAM, the infrared characteristic peaks of PAMAM and levofloxacin partially overlap. NHS-activated disulfide compounds exhibit an S–S vibration peak at 547 cm^–1^, a C–S vibration at 637 cm^–1^, a C–N stretching vibration at 1205 cm^–1^, a C–N stretching vibration of the amide III band at 1436 cm^–1^, a C=O stretching vibration of the amide I band at 1779 cm^–1^, and a C–H stretching vibration at 2989 cm^–1^. BSA exhibits an amide II band (involving N–H bending and C–N stretching vibrations) at 1541 cm^–1^, an amide I band at 1667 cm^–1^, C–H stretching vibrations at 2877 cm^–1^ and 2969 cm^–1^. In the levofloxacin-loaded BSA@PAMAM complex, weak S–S and C–S vibrations appear around 641 cm^–1^, N–H rocking vibrations appear at 800 and 975 cm^–1^, a C–F stretching vibration appears at 1289 cm^–1^, an asymmetric C–H bending vibration appears at 1459 cm^–1^ as well as an amide I band C=O stretching vibration, appears at 1624 cm^–1^. Comprehensive FTIR analysis indicates that the levofloxacin-loaded BSA@PAMAM complex exhibits characteristic peaks of levofloxacin while also incorporating the characteristic infrared peaks of BSA and PAMAM. These results confirm that levofloxacin has been successfully loaded into the PAMAM and that BSA has been successfully conjugated to the complex through a cross-linking agent, further validating the successful construction of this system.

**Figure 2:**
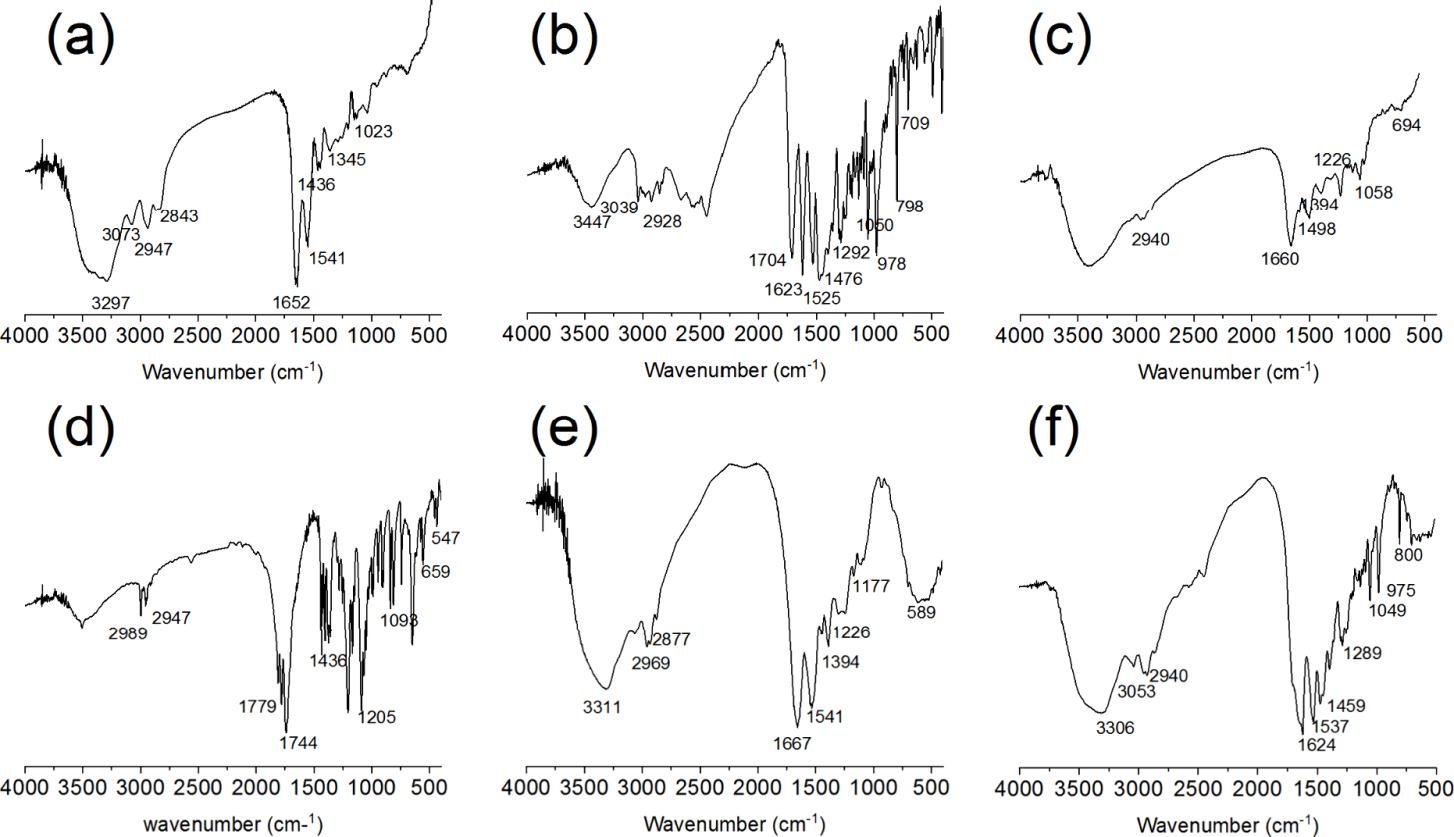
Analysis of PAMAM (a), levofloxacin (b), levofloxacin-loaded PAMAM (c), NHS-activated disulfide compound (d), BSA (e), and levofloxacin-loaded BSA@PAMAM (f).

The hydrophilicity and hydrophobicity of the BSA@PAMAM material were analyzed using contact angle measurements to assess its suitability as a nanodrug (Figure 3). For the PAMAM sample, the hydrophilic contact angle was measured at 50°, while the lipophilic contact angle was 32°, indicating that PAMAM exhibits weak hydrophilicity but relatively strong lipophilicity. This property arises from the molecular structure of PAMAM, a dendrimer with an ethylenediamine core that expands through iterative branching, forming an amphiphilic architecture conducive to drug loading and transport. The contact angle measurements for levofloxacin revealed a hydrophilic contact angle of 63° and a lipophilic contact angle of 51°, suggesting that the drug is predominantly hydrophobic. Upon loading levofloxacin onto PAMAM, the contact angles changed, with the hydrophilic contact angle increasing to 55° and the lipophilic contact angle increasing to 38°. This shift indicates that drug loading enhanced the overall hydrophobicity of the system, likely due to molecular interactions between levofloxacin and the PAMAM surface, which reduced its wettability in the aqueous phase. For BSA-modified materials, the contact angle results demonstrated strong hydrophilicity and weak lipophilicity, consistent with BSA’s intrinsic protein characteristics.^26^ After BSA was grafted onto PAMAM, the contact angle of the BSA@PAMAM nanodrug changed significantly. Compared to the unmodified PAMAM nanodrug (hydrophilic contact angle of 55°), the hydrophilic contact angle decreased substantially to 29° upon BSA modification, indicating that BSA significantly enhanced the hydrophilicity of the nanodrug. This result also confirms the successful modification of PAMAM with BSA. In summary, contact angle measurements validated the hydrophilic–lipophilic properties of the synthesized conjugated compounds. BSA-modified PAMAM exhibits superior amphiphilicity, which facilitates its dissolution and dispersion in aqueous environments while maintaining functionality in lipid-soluble conditions. This property suggests that BSA@PAMAM possesses excellent water solubility, making it a promising injectable antibiotic nanodrug and providing an experimental foundation for further drug delivery research.

**Figure 3:**
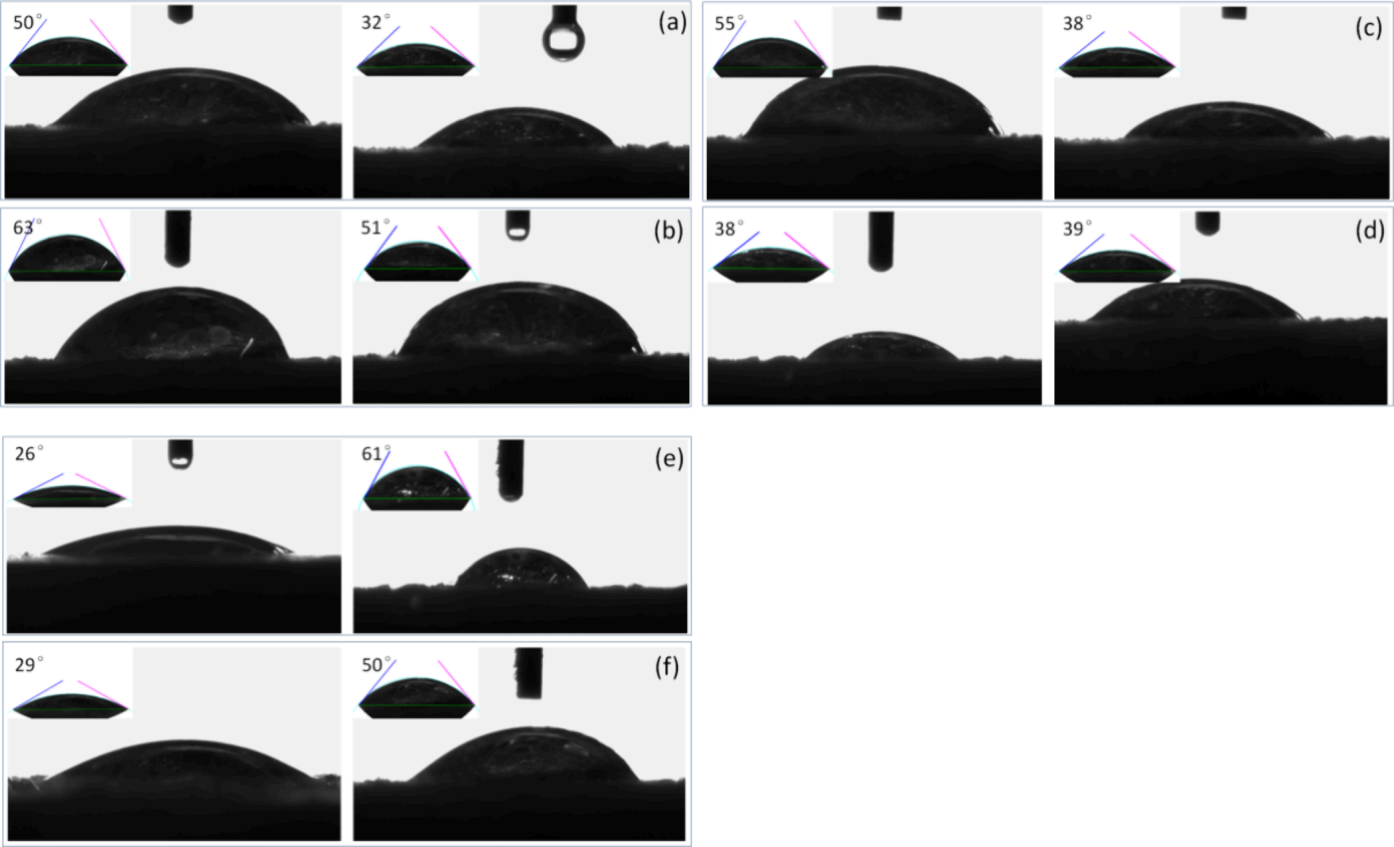
Hydrophilicity and hydrophobicity analysis of PAMAM (a), levofloxacin (b), levofloxacin-loaded PAMAM (c), NHS-activated disulfide compound (d), BSA (e), and levofloxacin-loaded BSA@PAMAM (f).

The hydrodynamic diameters of different samples in aqueous environments were measured using a nanoparticle size analyzer to verify size variations and assess their stability (Figure 4). The PAMAM exhibited a hydrodynamic diameter of approximately 100 nm with a polydispersity index (PDI) of 0.451, indicating the formation of stable nanoscale particles in water. In contrast, levofloxacin had a much smaller hydrodynamic diameter of around 50 nm (PDI 0.514), reflecting its inherently small molecular size. When levofloxacin was loaded onto PAMAM, the overall particle size increased slightly (PDI 0.357), but the change was not significant, suggesting that drug encapsulation had a limited impact on the hydrodynamic size of PAMAM. For the NHS-activated disulfide compound, the hydrodynamic diameter in water was significantly smaller, measuring around 10 nm (PDI 0.391). This may be attributed to its high amphiphilicity and low molecular weight, allowing it to maintain a compact structure in aqueous solutions. The BSA molecule exhibited a hydrodynamic diameter of approximately 120 nm (PDI 0.582), whereas the BSA-modified PAMAM composite displayed a significantly larger particle size. This increase confirms the successful conjugation of BSA onto the PAMAM surface, forming a larger composite structure. Furthermore, the levofloxacin-loaded BSA@PAMAM composite exhibited a further increase in particle size (PDI 0.573). This could be due to the successful encapsulation of levofloxacin within the PAMAM structure, along with the formation of a stable coating layer by BSA on the PAMAM surface. These variations in particle size further support the efficient conjugation of BSA to PAMAM, which enhances the stability of the nanodrug and facilitates its application in aqueous environments. We have observed that the PDI values of the nanoparticles prepared in the current stage are generally high. This is primarily attributed to the inherent properties of PAMAM and BSA, which are prone to aggregation through electrostatic interactions or hydrophobic forces, leading to a broad size distribution in the aqueous phase. These data objectively reveal the limitations of the current preparation process and clearly indicate that in subsequent studies, we need to focus on optimizing the formulation and purification processes to improve the monodispersity of the nanoparticles. In summary, the particle size measurements confirmed the successful loading of levofloxacin and the effective modification of PAMAM by BSA, providing experimental evidence for BSA@PAMAM as a stable drug delivery system.

**Figure 4:**
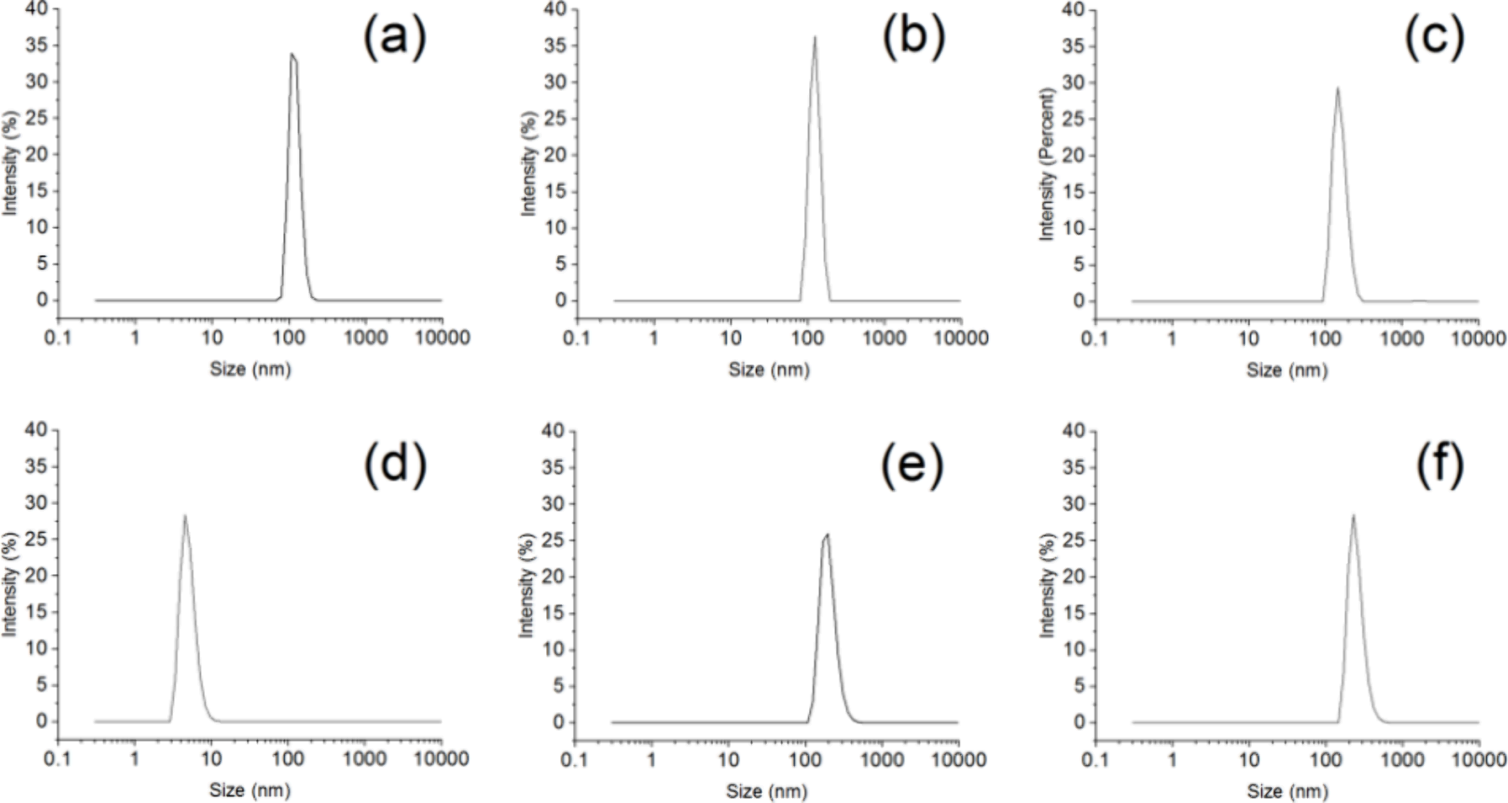
Hydrodynamic size analysis of PAMAM (a), levofloxacin (b), levofloxacin-loaded PAMAM (c), NHS-activated disulfide compound (d), BSA (e), and levofloxacin-loaded BSA@PAMAM (f).

To further verify the successful preparation of levofloxacin-loaded BSA@PAMAM, thermogravimetric analysis (TGA) was performed on PAMAM, levofloxacin, levofloxacin-loaded PAMAM, the coupling agent, BSA, and levofloxacin-loaded BSA@PAMAM (Figure 5). For PAMAM, the thermal degradation process can be divided into two stages. In the range of room temperature to 100 °C, the mass loss was minimal. From 100 to 260 °C, the weight loss was approximately 12%. Subsequently, in the range of 260 to 400 °C, the weight loss reached 80%, which is likely related to the decomposition of the PAMAM framework. For levofloxacin, thermal degradation started at around 300 °C, and by 400 °C, the weight loss was approximately 55%, indicating a relatively slow decomposition at higher temperatures. For levofloxacin-loaded PAMAM, the material exhibited slow weight loss between room temperature and 250 °C, which may be attributed to the evaporation of adsorbed water. A significant weight loss was observed between 250 and 400 °C, which could be associated with the decomposition of the PAMAM framework. By 400 °C, the weight loss tended to stabilize, suggesting that PAMAM successfully loaded levofloxacin and reached a thermal equilibrium state at high temperatures. For the NHS-activated disulfide compound, thermal degradation mainly occurred around 300 °C, where the weight loss rate increased rapidly, indicating that the coupling agent decomposed easily at this temperature and reached its maximum mass loss, demonstrating poor thermal stability. For BSA, thermal degradation also occurred in two stages. In the range of 220 to 350 °C, the weight loss rate was relatively fast, with a mass loss of approximately 55%, which could be related to the evaporation of bound water, hydrogen bond disruption, and amino acid degradation. Subsequently, in the range of 350 to 700 °C, BSA exhibited a slow and continuous weight loss, which may be attributed to the gradual decomposition of its internal structure. For levofloxacin-loaded BSA@PAMAM, due to the presence of the NHS-activated disulfide compound and BSA, the major weight loss occurred between 200 and 400 °C. As the temperature increased to 700 °C, the weight loss trend became more stable, resembling the thermal degradation behavior of PAMAM. This result indicates that BSA was successfully loaded onto levofloxacin-loaded PAMAM via the coupling agent, thereby enhancing the hydrophilicity and stability of the nanodrug’s outer layer. In summary, the thermogravimetric analysis results fully confirm the successful preparation of BSA@PAMAM and further demonstrate that the nanodrug exhibits good thermal stability, which contributes to its controlled drug release capability and application potential in physiological environments.

**Figure 5:**
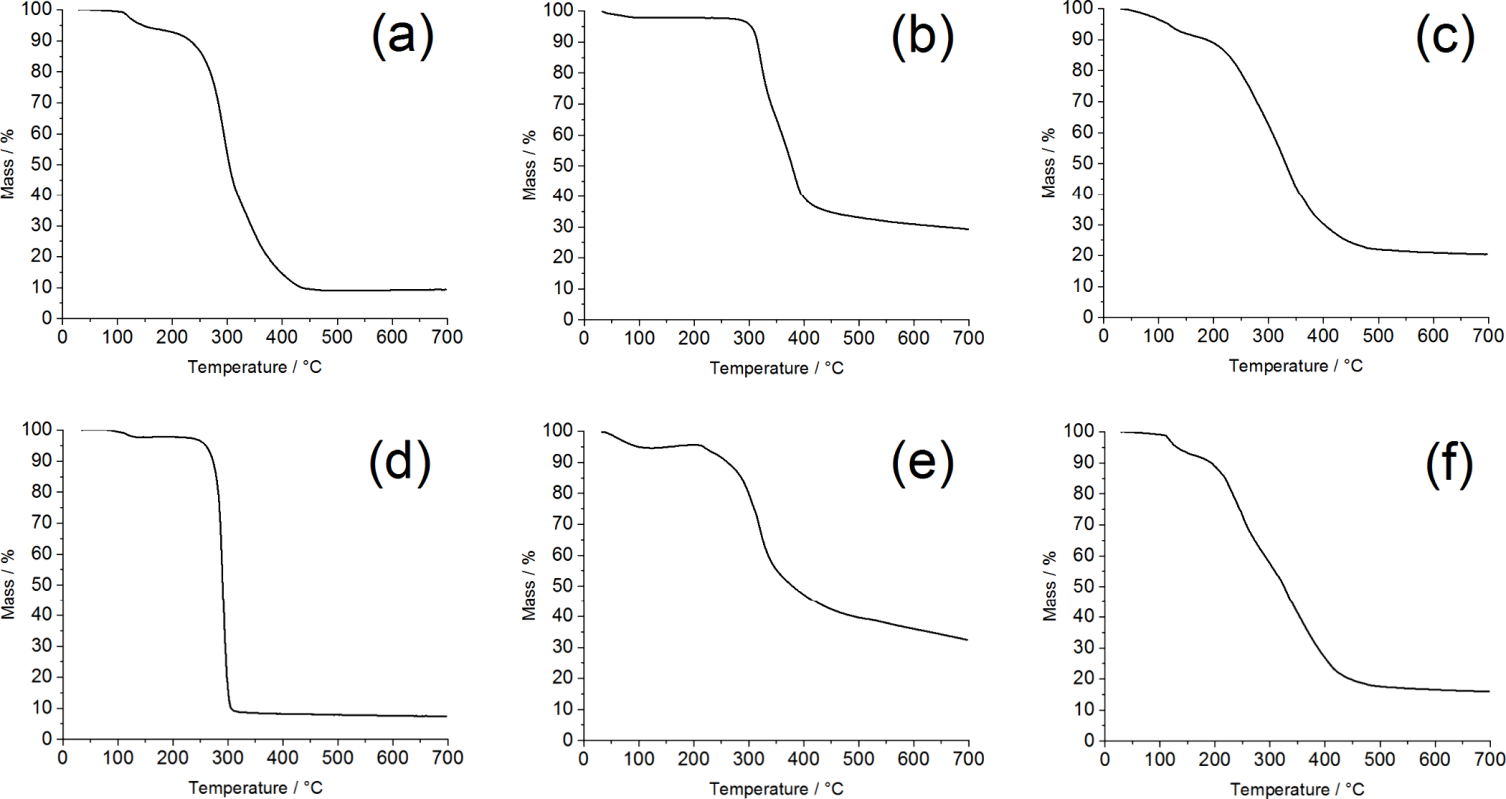
Thermogravimetric analysis of PAMAM (a), levofloxacin (b), levofloxacin-loaded PAMAM (c), NHS-activated disulfide compound (d), BSA (e), and levofloxacin-loaded BSA@PAMAM (f).

### 3.2 Drug Loading and Release Behavior of Levofloxacin-Loaded BSA@PAMAM

The drug loading capacity of BSA@PAMAM is 15.2%. To objectively assess this performance, we compared it with several carriers reported in the literature. Among them, hydroxypropyl-β-cyclodextrin and metal–organic frameworks@silk composites have relatively high drug loading capacities, at 17.4%^29^ and 20%,^30^ respectively. In contrast, mesoporous silica nanoparticles decorated with polycationic dendrimers and PLGA have relatively lower drug loading capacities, at 7.8%^31^ and 10%,^32^ respectively. From this comparison, it can be seen that BSA@PAMAM has a drug loading capacity that ranks above average in the above systems. Although it does not reach the highest values, it still demonstrates considerable drug loading capacity, indicating that this carrier has competitive potential in terms of drug loading performance. Figure 6 illustrates the in vitro drug release behavior of the levofloxacin-loaded BSA@PAMAM system in different solution environments. When using physiological saline to simulate normal body fluids, the drug release rate remained extremely slow during the 0–50 h period, indicating that the nanodrug effectively inhibited the nonspecific release of the drug. In contrast, in physiological saline containing 5 mM GSH and 10 mM GSH, the release rate of levofloxacin increased significantly and the release rate in the 10 mM MGSH solution is faster than that in the 5 mM GSH solution, especially within the 0–20 h period, where the drug was rapidly released into the simulated GSH solution. Additionally, we conducted mathematical simulations on levofloxacin-loaded BSA@PAMAM to further explain the potential release mechanism of BSA@PAMAM. The Korsmeyer-Peppas, Higuchi, and First-order models were used to fit the release behavior of levofloxacin (Figure 6 and Table 1). Among the three fitting models, the First-order model had the highest *R* ^2^ value, indicating that BSA@PAMAM, when stimulated by GSH, undergoes disulfide bond cleavage, releasing levofloxacin. Initially, the drug concentration is high, and the release rate is fast; as the drug continues to be released, the drug concentration within the carrier decreases, and the release rate slows down. At the same time, based on the *R* ^2^ value (*R* ^2^ ≥ 0.8), the drug release behavior of this carrier also closely follows the Korsmeyer-Peppas and Higuchi models, suggesting that the release of levofloxacin from PAMAM is in line with the typical process of drug release from a carrier (Table 1). According to the Korsmeyer-Peppas model, the release exponent (*n*) is 0.6 (>0.5), indicating that the release of levofloxacin is controlled by non-Fickian diffusion.^33^ There are relatively strong intermolecular interactions (including hydrophobic interactions, hydrogen bonding, etc.) between levofloxacin and PAMAM, and the albumin coating further hinders the diffusion of levofloxacin to the external environment.^34^ This phenomenon demonstrates the GSH-responsive properties of the BSA@PAMAM nanodrug. These results indicate that conjugating BSA to the levofloxacin-loaded PAMAM nanodrug via a disulfide-containing compound effectively seals the drug, preventing its nonspecific release under normal physiological conditions. However, in the presence of GSH, the disulfide bonds are cleaved, triggering the controlled release of the drug. Therefore, using NHS-activated disulfide compounds to conjugate BSA onto the PAMAM nanodrug not only enhances its stability but also successfully achieves a GSH-triggered drug release mechanism. This design strategy holds great potential for achieving precise drug delivery in GSH-rich microenvironments.

**Figure 6:**
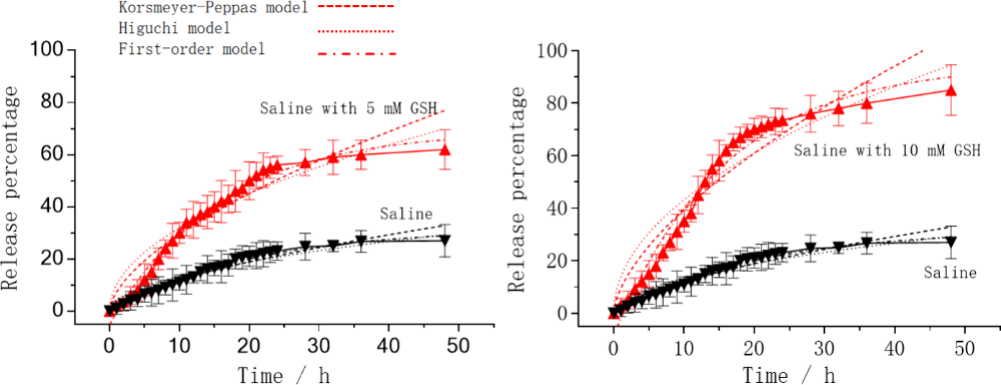
Drug release behavior of levofloxacin-loaded BSA@PAMAM in normal saline and normal saline with 5 mM GSH and 10 mM GSH and fitted by Korsmeyer–Peppas, Higuchi, and first-order models.

**Table 1:** Kinetic Models for Levofloxacin Release from BSA@PAMAM

	release kinetics model					
groups	Korsmeyer– Peppas		Higuchi		first-order	
10 mM GSH	*Q* = 9.3038*t ^0.6272^	*R* ^2^ = 0.80	*Q* = 13.65*t ^0.5^	*R* ^2^ = 0.85	*Q* = 86.58*(1 – e^–0.06398^)	*R* ^2^ = 0.9526
5 mM GSH	*Q* = 7.234*t ^0.6107^	*R* ^2^ = 0.88	*Q* = 10.09*t ^0.5^	*R* ^2^ = 0.86	*Q* = 63.902*(1 – e^–0.06308^)	*R* ^2^ = 0.9405
saline	*Q* = 2.83*t ^0.6332^	*R* ^2^ = 0.79	*Q* = 4.227*t ^0.5^	*R* ^2^ = 0.78	*Q* = 32.78*(1 – e^–0.05225^)	*R* ^2^ = 0.8399

### 3.3 Cytotoxicity of BSA@PAMAM Delivery System

To evaluate the cytotoxicity of the blank nanodrug on normal cells (HEK293T), we assessed cell viability using the CCK-8 assay (Figure 7). In the experiment, HEK293T cells were coincubated with different concentrations of the blank nanodrug for 24 h. The results showed that even at a relatively high concentration (700 μg/mL), the cell viability remained above 80%, indicating that the blank nanodrug exhibited low cytotoxicity toward normal cells. This finding suggests that the prepared drug nanodrug possesses good biocompatibility at high concentrations and does not significantly affect the proliferation and survival of normal cells. Furthermore, this provides a preliminary basis for its safety in subsequent drug delivery applications.

**Figure 7:**
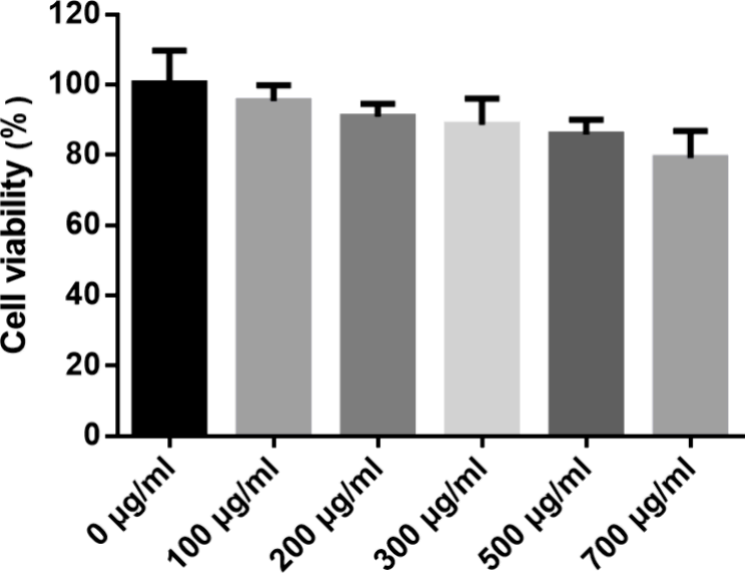
Effect of drug-free BSA@PAMAM on cell viability.

### 3.4 Antibacterial Assay of Levofloxacin-Loaded BSA@PAMAM Compared to Levofloxacin

To verify the antibacterial efficacy of the levofloxacin-loaded BSA@PAMAM nanodrug, we conducted a comparative analysis using different experimental groups, including the levofloxacin group, the levofloxacin-loaded BSA@PAMAM nanodrug group and the blank nanodrug group, (Figure 8). The experiments were performed with different concentrations and incubation media (normal saline and normal saline +5 mM GSH) to investigate the antibacterial mechanism and release characteristics of the nanodrug system. For the levofloxacin group, the inhibition zone continuously increased with the increasing levofloxacin concentration across different incubation media. Moreover, levofloxacin demonstrated a relatively ideal antimicrobial effect even at low concentrations, suggesting that its antibacterial effect primarily depends on the drug’s diffusion and direct bactericidal action rather than being influenced by GSH or the carrier environment. This also indicates that levofloxacin has a rapid release rate, potentially leading to a high initial drug concentration, which may cause adverse side effects. For the levofloxacin-loaded BSA@PAMAM nanodrug group, a clear concentration-dependent antibacterial effect was observed, when incubated in GSH environment, with larger inhibition zones at higher concentrations. Even at a low drug concentration of 20 μg/mL, anobvious inhibition zone was still observed. However, in saline environment, levofloxacin-loaded BSA@PAMAM only showed a noticeable antimicrobial effect at relatively high concentrations (70 μg/mL). Moreover, in the incubation medium containing 5 mM GSH, the antibacterial efficacy of this group was significantly enhanced, demonstrating the GSH-responsive properties of the BSA@PAMAM nanodrug. In the presence of GSH, the drug release rate increased, enabling a more controlled release in the target environment, which helps improve drug bioavailability while reducing the toxicity risk associated with nontargeted release. For the blank nanodrug group, the inhibition zones were relatively smallor none under different concentrations and incubation conditions, indicating that BSA@PAMAM itself does not exhibit significant antibacterial activity and that the nanodrug material does not substantially affect bacterial growth. Based on the antimicrobial effect of levofloxacin-loaded BSA@PAMAM nanodrug at different concentrations against *Escherichia coli*, we then used 50 μg/mL of levofloxacin-loaded BSA@PAMAM to observe its antimicrobial effect in both saline and GSH environments (Figure 9). The results of the dead bacteria staining experiment revealed a clear time-dependent antibacterial effect following coincubation of the bacterial suspension with levofloxacin-loaded BSA@PAMAM, which was strongly regulated by the environmental GSH concentration. In a saline environment, only weak red fluorescence was observed, indicating that the carrier system remained stable with minimal drug release. However, under GSH conditions simulating the intracellular environment, the intensity of red fluorescence increased significantly over time, clearly indicating that a large amount of levofloxacin was released from BSA@PAMAM, leading to bacterial death. This dynamic bactericidal process was highly consistent with the trend observed in the inhibition zone by the agar diffusion method, providing strong evidence for the GSH-responsive drug release mechanism of this drug delivery system and its efficient antibacterial efficacy. Over time, levofloxacin-loaded BSA@PAMAM showed an increasingly pronounced antimicrobial effect in the GSH environment, whereas in the saline environment, the antimicrobial effect of levofloxacin-loaded BSA@PAMAM remained less noticeable. This indicates that the antimicrobial effect of levofloxacin-loaded BSA@PAMAM exhibits a clear GSH-responsive behavior. Repeated experiments showed good consistency in the antibacterial results under identical conditions across all experimental groups, further confirming the reliability of the study. The core of this design lies in utilizing the GSH-responsive nature of disulfide bonds to achieve controlled drug release. To this end, we selected NHS-activated disulfide compounds as functional linkers, which feature both disulfide bonds and NHS active esters. On one hand, the NHS active ester can efficiently react with the abundant amine groups on the surfaces of PAMAM and BSA to form a stable BSA coating structure, significantly enhancing the stability of the drug-loaded system in physiological environments.^28^ On the other hand, the disulfide bonds introduced into the structure serve as specific GSH-responsive sites, enabling rapid and specific drug release in the intracellular environment due to the significant GSH concentration difference between the inside (1–10 mM) and outside (∼50 μM) of the cell.^11^ This responsive release mechanism has been supported by several previous studies,^35^ further validating the rationality and reliability of the design strategy for this carrier. In conclusion, the BSA@PAMAM nanodrug not only achieves a sustained release effect for levofloxacin but also enhances drug release under GSH stimulation, exhibiting good environmental responsiveness and targeted drug delivery capability. These characteristics improve antibacterial efficacy while minimizing side effects, highlighting its potential application value in antimicrobial therapy.

**Figure 8:**
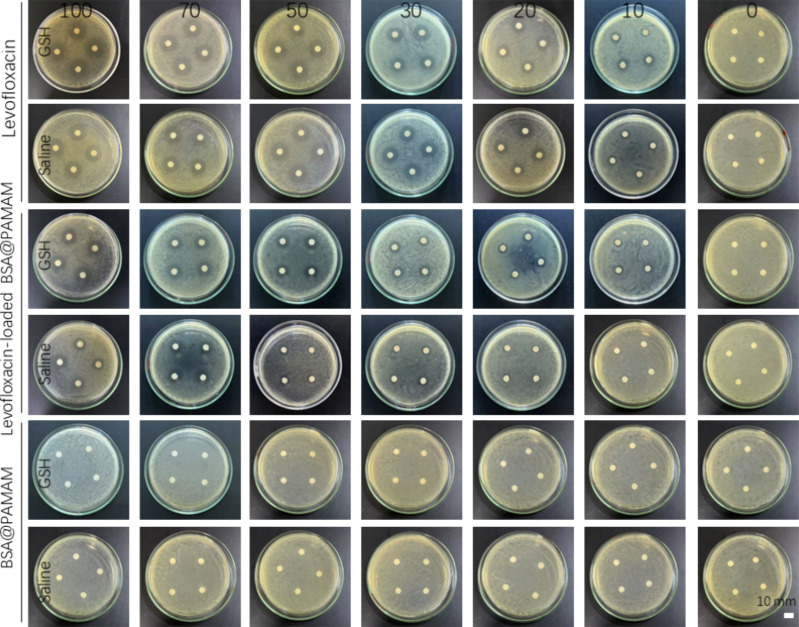
Inhibitory effects of levofloxacin, levofloxacin-loaded BSA@PAMAM, and drug-free BSA@PAMAM on *Escherichia coli*. Concentrations: 100, 70, 50, 30, 20, 10, 0 μg/mL. Note: The drug concentration of each group was based on the amount of levofloxacin.

**Figure 9:**
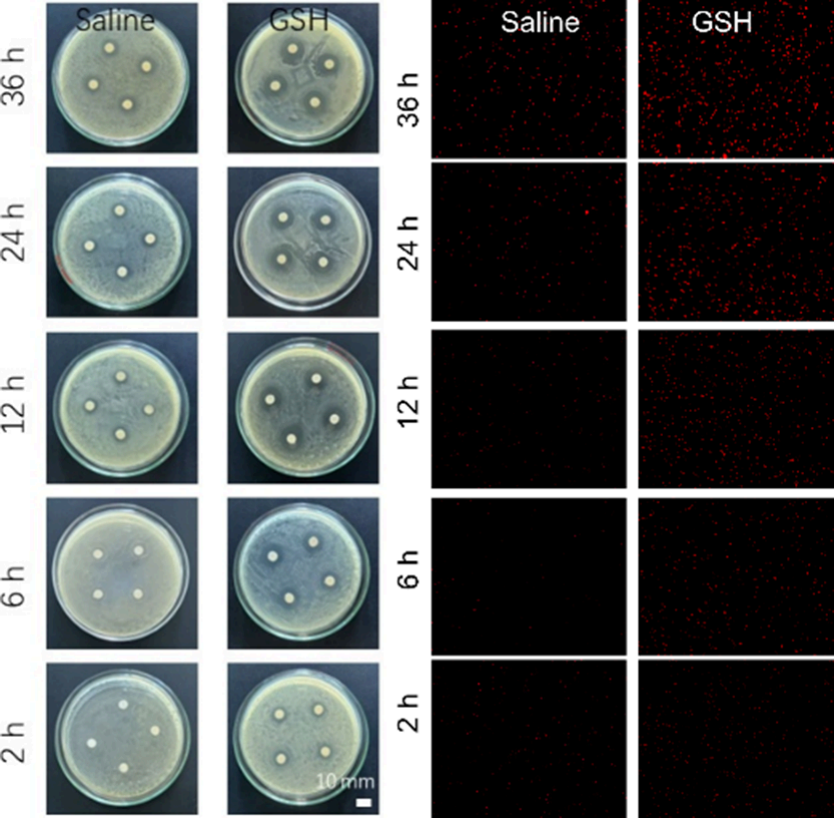
Inhibitory effects of levofloxacin-loaded BSA@PAMAM on *Escherichia coli* at different times in saline and GSH environments, respectively. Concentration: 50 μg/mL. Note: The drug concentration of each group was based on the amount of levofloxacin. Left: plate culture analysis, right: suspension culture analysis.

Significant progress has been made in the surface functionalization of PAMAMs, with related strategies continuously optimized and upgraded. For example, PAMAM modified with cholesterol can improve targeting ability. Using organic silane coupling agents to combine Fe_3_O_4_@SiO_2_ with PAMAMs can impart magnetic targeting properties.^36^ The introduction of boronic acid molecules with PAMAM can enhance their ability to recognize Gram-positive/negative bacteria,^37^ and PEGylation of the PAMAM surface helps extend its circulation time in vivo, reducing immune cell recognition and clearance.^38^ In this study, we used PAMAM dendrimers as the carrier for levofloxacin. This molecule features internal cavities and abundant surface functional groups, which allow the efficient encapsulation of drug molecules through physical entrapment and electrostatic interactions, forming water-soluble nanocomposites. This significantly enhances the solubility of levofloxacin in bodily fluids. Additionally, the drug loaded within the PAMAM is well-protected, preventing premature degradation by enzymes or chemical inactivation in the body, further improving its stability. Traditional administration of levofloxacin is typically systemic, resulting in widespread distribution of the drug, with only a small amount reaching the infection site. To achieve an effective therapeutic concentration, high doses are often required, leading to systemic toxic side effects.^6,7^ To address this, we introduced a strategy combining BSA with PAMAM, effectively providing the system with a “biomimetic camouflage”.^21,39^ This design cleverly integrates the advantages of both materials, significantly reducing the probability of immune system recognition and phagocytosis, prolonging the nanodrug’s half-life in the bloodstream, and laying the foundation for precise and efficient drug delivery.

## 4 Conclusions

This study successfully utilized the hydrophobic cavity of PAMAM to efficiently load levofloxacin and employed a coupling strategy to coat albumin on the outer surface of PAMAM, rendering the nanodrug hydrophilic. This design resulted in a stable BSA@PAMAM nanodrug with GSH-responsive drug release properties. The successful synthesis and structural modifications of the material were validated through multiple characterization techniques: ^1^H NMR and ^13^C NMR confirmed the successful synthesis of NHS-activated esters and disulfide derivatives. FTIR spectroscopy further verified the stepwise modification of the levofloxacin-loaded BSA@PAMAM. Contact angle measurement, DLS, and TGA were used to assess changes in hydrophilicity, particle size evolution, and thermal stability at different modification stages, aligning with the expected characteristics of nanodrug throughout the modification process. Notably, drug release experiments demonstrated that the BSA@PAMAM nanodrug exhibited strong GSH responsiveness, enabling controlled drug release in the presence of GSH and enhancing drug delivery efficiency in the targeted environment. Biocompatibility studies confirmed that the nanodrug exhibited minimal toxicity, while antibacterial experiments further demonstrated its effective controlled release in a GSH-enriched environment, improving the bioavailability of levofloxacin while reducing unnecessary systemic toxicity. To realize the clinical translation potential of BSA@PAMAM, subsequent research will focus on the following aspects: First, in vivo pharmacodynamic evaluation will be conducted using an infection mouse model to verify its actual therapeutic effect. Second, a systematic study will be performed on the in vivo distribution, pharmacokinetic behavior, and long-term safety of the carrier to provide critical data support for clinical applications. Finally, the formulation process will be further optimized to ensure the stability and reproducibility of the carrier. In conclusion, the BSA@PAMAM nanodrug designed in this study exhibits excellent stability, hydrophilicity, GSH-responsive drug release properties, and good biocompatibility. With its superior controlled release capabilities, this nanodrug holds significant potential for application in intelligent drug delivery systems.

## Supplementary Material



## Data Availability

The data supporting this study are available within the manuscript.

## Notes

Standard curve of levofloxacin (PDF)
