# Supplementary material for: Bovine Serum Albumin-Functionalized Hyperbranched Polyamidoamine Dendrimers (BSA@PAMAM) for GSH-Responsive Bacteriostasis
Source: ACS Omega. 2026 Jan 16;11(4):5579–91. doi: 10.1021/acsomega.5c09261 (PMC12878411; doi:10.1021/acsomega.5c09261)
Supplement: Supplementary file 1 [file ao5c09261_si_001.pdf]

Bovine Serum Albumin-Functionalized Hyperbranched Polyamidoamine Dendrimers (BSA@PAMAM) for GSH-responsive Bacteriostasis

Yu Fu [a], Yixuan Ren [b], Shufen Xiao [b], Jian Chen [b], Xingling Liu [a,*]

[a] Department of Pharmacy, Third Affiliated Hospital of Sun Yat-sen University, Guangzhou 510630, China

[b] School of Chemistry and Chemical Engineering, Hunan University of Science and Technology, Xiangtan, Hunan, 411201, People's Republic of China

[*] Corresponding author. Department of Pharmacy, Third Affiliated Hospital of Sun Yat-sen University, Guangzhou 510630, China

E-mail: liuxl55@mail.sysu.edu.cn

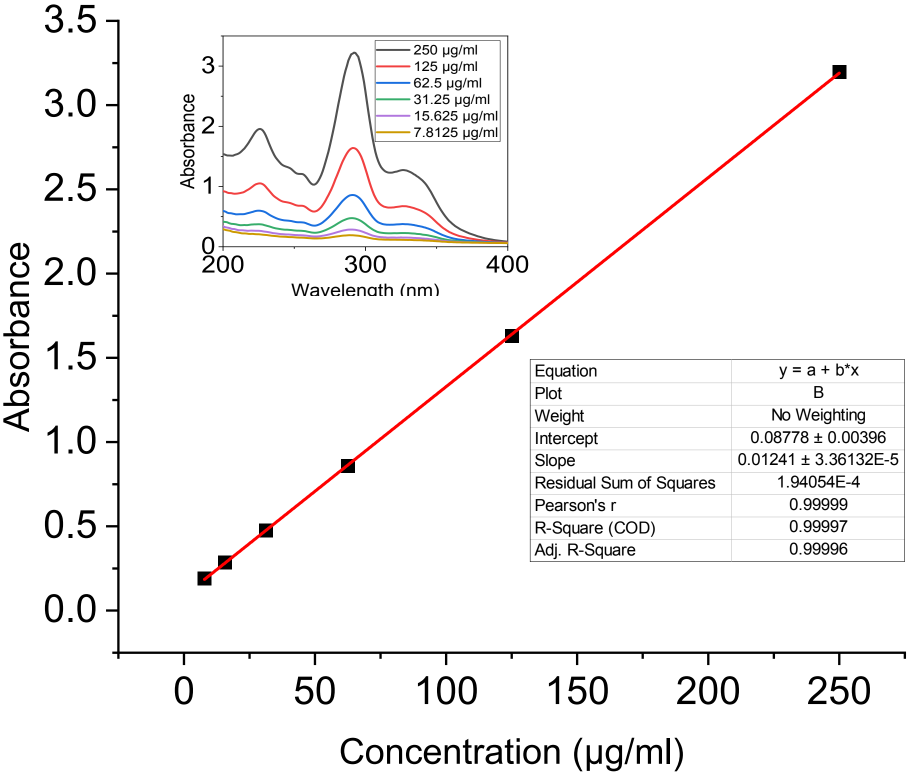

Figure S1. The standard curve of levofloxacin
